# Interleukin-1*β* Affects MDAMB231 Breast Cancer Cell Migration under Hypoxia: Role of HIF-1*α* and NF*κ*B Transcription Factors

**DOI:** 10.1155/2015/789414

**Published:** 2015-11-30

**Authors:** Irene Filippi, Fabio Carraro, Antonella Naldini

**Affiliations:** Department of Molecular and Developmental Medicine, Cellular and Molecular Physiology Unit, University of Siena, 53100 Siena, Italy

## Abstract

Inflammation and tumor hypoxia are intimately linked and breast cancer provides a typical example of an inflammation-linked malignant disease. Indeed, breast cancer progression is actively supported by inflammatory components, including IL-1*β*, and by the hypoxia-inducible factor- (HIF-) 1*α*. In spite of many attempts where the role of either IL-1*β* or HIF-1*α* was evaluated, detailed mechanisms for their effects on breast cancer cell migration under hypoxia are still unclear. We here report that IL-1*β* increased MDAMB231 cell migration under hypoxic conditions along with HIF-1*α* accumulation and upregulation of CXCR1, which is transcriptionally regulated by HIF-1*α*, as well as an increased expression of CXCL8 and NF*κ*B. In addition, IL-1*β*-induced cell migration in hypoxia was not affected when HIF-1*α* was inhibited by either siRNA or Topotecan, well known for its inhibitory effect on HIF-1*α*. Of interest, HIF-1*α* inhibition did not reduce NF*κ*B and CXCL8 expression and the reduction of IL-1*β*-induced cell migration under hypoxia was achieved only by pharmacological inhibition of NF*κ*B. Our findings indicate that inhibition of HIF-1*α* does not prevent the migratory program activated by IL-1*β* in hypoxic MDAMB231 cells. They also suggest a potential compensatory role of NF*κ*B/CXCL8 pathway in IL-1*β*-induced MDAMB231 cell migration in a hypoxic microenvironment.

## 1. Introduction

The link between inflammation and cancer is an old concept, firstly proposed by Virchow in 1863 [1]. The tumor microenvironment, hypoxia, and inflammation are critical components necessary for tumor progression and for the metastatic cascade [2]. Indeed, when compared with a normal condition, such environment is more permissive of tumor cell proliferation and motility. Furthermore, studies conducted in intravital mouse models and* in vitro* indicate that tumor cell signaling and extracellular signaling cues influence cancer cell migration and therefore metastasis [3].

Breast cancer provides a typical example of an inflammation-linked malignant disease associated with a hypoxic microenvironment [4] and its progression is actively supported by inflammatory components, including proinflammatory cytokines [5].

Interleukin- (IL-) 1*β* is the prototypical proinflammatory cytokine [6] and its expression in most tumors correlates with tumor invasiveness and metastasis, as well as with angiogenesis [7, 8]. Several studies have shown how IL-1*β* may contribute to breast cancer development and metastasis [9, 10]. Among its multiple effects, IL-1*β* activates a hypoxia-angiogenesis program by upregulating the hypoxia-inducible factor- (HIF-) 1*α*, the pivotal mediator of cellular responses to hypoxia [11]. HIF-1*α* is a basic helix-loop-helix-PER-ARNT-SIM (bHLH-PAS) protein that forms heterodimeric complexes composed of an O_2_-labile *α*-subunit and a stable *β*-subunit (also known as ARNT), which bind together to the hypoxia responsive elements (HREs), located in the promoter of target genes [12]. HIF-1*α* expression in cancers is associated with clinical aggressiveness and poor outcome [4]. We and others have previously demonstrated that IL-1*β* upregulated HIF-1*α* protein under normoxia in breast, lung, and colon cancer cells, using either a posttranscriptional mechanism or a pathway dependent on nuclear factor *κ*B (NF*κ*B) [11–13]. The latter belongs to a family of transcription factors, typically associated with inflammation, that are very frequently activated in tumors and involved in tumor growth, progression, and resistance to chemotherapy [14–16]. NF*κ*B exists as a heterodimer composed of RelA (p65) and NF*κ*B1 (p50) subunits in most cells, where it regulates the expression of multiple genes and contributes to the pathogenesis of various diseases, including breast cancer [17–19]. Upon activation by various stimuli, including IL-1*β*, NF*κ*B transcriptionally regulates several genes involved in early inflammatory responses, such as the chemokine CXCL8 [20], in a variety of cells, including breast cancer cells [10]. Importantly, CXCL8 plays a critical role in the increased migratory activity of different human breast carcinoma cell lines under oxygen deprivation [21].

Given the relevance of the interplay between inflammation and hypoxia in tumor progression, in this study we decided to investigate the role of HIF-1*α* and NF*κ*B in MDAMB231 breast cancer cell migration under hypoxic conditions and in the presence of IL-1*β*.

## 2. Materials and Methods

### 2.1. Reagents and Cell Culture

Recombinant human IL-1*β* and BAY 11-7082 were purchased from Pierce Endogen (Rockford, IL) and from Tocris Bioscience (Bristol, UK), respectively. MDAMB231 breast cancer cell line and Topotecan were kindly provided by Dr. Raffaella Giavazzi (IRCCS-Istituto di Ricerche Farmacologiche Mario Negri Milano, Italy). MDAMB231 cells were cultured in RPMI 1640 (Euroclone, Devon, UK), supplemented with 10% FBS (Euroclone) and cultured in atmospheric O_2_ tension (20% ~140 mmHg), 5% CO_2_, and 90% humidity at 37°C. The experiments under hypoxia were performed using the workstation INVIVO_2_ 400 (Ruskinn, Pencoed, UK) providing a customized and stable humidified (90%) environment at 37°C through electronic control of O_2_ (hypoxia: 2% ~14 mmHg) and CO_2_ (5%) [22]. Such conditions resemble a mild hypoxic state similar to that observed in breast cancer tissues* in vivo* [23].

### 2.2. Migration Assay

MDAMB231 cell migration was evaluated by a commercially available Transwell System (Corning Costar, Cambridge, MA) and performed in 6.5 mm diameter chambers with pore size of 8.0 *μ*m. Serum-starved cells (1 × 10^6^ cells/mL) were resuspended in DMEM supplemented with 0.5% bovine serum albumin (BSA) and added to the upper chamber and a solution of medium supplemented with 0.5% BSA was added to the lower chamber. Where indicated, IL-1*β*, Topotecan, or BAY 11-7082 was added to the upper chamber. After 24 hours of hypoxic incubation (2% O_2_), cells on the upper membrane surface were removed by careful wiping with a cotton swab. The number of migrating cells was determined by CyQuant DNA binding fluorescence according to manufacturer's instructions (Molecular Probes, Thermo Fisher Scientific, Waltham, MA) [24]. Relative fluorescence was detected at 485 nm excitation and 520 nm emission by fluorescence microplate reader FLUOstar OPTIMA (BMG LABTECH, Durham, NC). Results were expressed as migration index, calculated as a fold change of relative CyQuant fluorescence of cells migrating into the lower chamber normalized to untreated control conditions.

Cell migration was also evaluated in parallel experiments, by using modified Boyden 48-well micro chemotaxis chambers (Neuro Probe, Gaithersburg, MD) with 8 *μ*m pore size polycarbonate polyvinylpyrrolidone-free Nucleopore filters as previously described [13]. After 6 hours of incubation under hypoxia, cells on the upper surface of the filter were removed. Migrating cells on the lower surface were stained using Diff-Quick (Merz-Dade, Düdingen, CH) and photographed by an OLYMPUS IX81 Research Microscope with a 10x magnification. The images were processed with software Soft Imaging System (Olympus Company, Munster, D). Cells migrating in 10 high-power fields were counted and data were expressed as number of migrating cells/field. Each assay was done in quadruplicate.

To rule out the possibility that the results may be affected by either cell proliferation or cell death, cell viability was routinely assessed by LIVE/DEAD Viability/Cytotoxicity Assay (Molecular Probes, Thermo Fisher Scientific, Waltham, MA), as previously described [25].

### 2.3. Preparation of Cell Extracts and Western Blotting

MDAMB231 cells were seeded in 6-well plates at 1.5 × 10^5^ cells per well. After an overnight serum-starvation, cells were treated with IL-1*β* in the presence or in the absence of Topotecan or BAY 11-7082 and incubated under hypoxic conditions (2% O_2_) for 8 hours, unless specifically described. Thereafter cells were lysed and equal amounts of proteins from whole cell extracts were subjected to SDS-PAGE followed by protein transfer to a PVDF membrane, as previously reported [26]. Protein analysis was performed by Western blotting using antibodies against HIF-1*α* (BD Biosciences, San Jose, CA), phospho-NF*κ*B p65 (Ser536), phospho-p38 MAPK, or *β*-actin (Cell Signaling, Danvers, MA). Chemiluminescence was quantified using a ChemiDoc XRS apparatus and Quantity One software (Bio-Rad Laboratories, Hercules, CA).

### 2.4. mRNA Detection by qRT-PCR

Total RNA was extracted from hypoxic cells using TRI Reagent following the manufacturer's instructions (Ambion, Thermo Fisher Scientific, Waltham, MA), as previously reported [27]. cDNA was synthesized using iScript cDNA Synthesis Kit (Bio-Rad Laboratories, Hercules, CA). qRT-PCR was performed using ITaq SYBR Green Supermix with ROX (Bio-Rad Laboratories, Hercules, CA). The primers were designed using the PRIMER3 program (available at http://frodo.wi.mit.edu) and are shown in Table 1.

Thermocycler conditions included an initial holding at 95°C for 3 minutes; this was followed by a 2-step PCR program: 95°C for 15 seconds and 60°C for 20 seconds for 49 cycles. Data were quantitatively analyzed on an iQ 5 Multicolor Real-Time PCR Detection System (Bio-Rad Laboratories, Hercules, CA). Relative quantification was done by using the 2^−ΔΔCT^ method, as previously described [28]; *β*-actin was used as housekeeping gene and results were expressed relatively to the control cells.

### 2.5. siRNA Transfection

siRNA sequences were selected according to Invitrogen stealth RNA system (Invitrogen, Thermo Fisher Scientific, Waltham, MA) for specific silencing of HIF-1*α*. The siRNA target sequences correspond to nucleotides 1028 to 1049 (NM_001530) of human HIF-1*α* mRNA; the negative controls were designed with the same CG ratio without any known target to the human genome, as previously described [26]. Cells were transfected by using Lipofectamine (Invitrogen, Thermo Fisher Scientific, Waltham, MA) according to the manufacturer's instructions. Following transfection, cells were exposed to hypoxia and cell migration, Western blot, and qRT-PCR analysis were conducted, as described above.

### 2.6. ELISA

CXCL8 and VEGF concentrations were assessed on cell-free supernatants by ELISA using commercial high-performance ELISA reagents (R&D Systems, Minneapolis, MN). The assay did not show cross-reactivity with other cytokines. The minimum detectable dose for CXCL8 was <25 pg/mL.

### 2.7. Statistical Analysis

Values presented are the means ± SD of the results obtained from at least three independent experiments, unless specifically described. Statistical significance between means was determined using Student's *t*-test. 

## 3. Results 

### 3.1. IL-1*β* Increases MDAMB231 Cell Migratory Ability under Hypoxic Conditions

**Figure 1:**
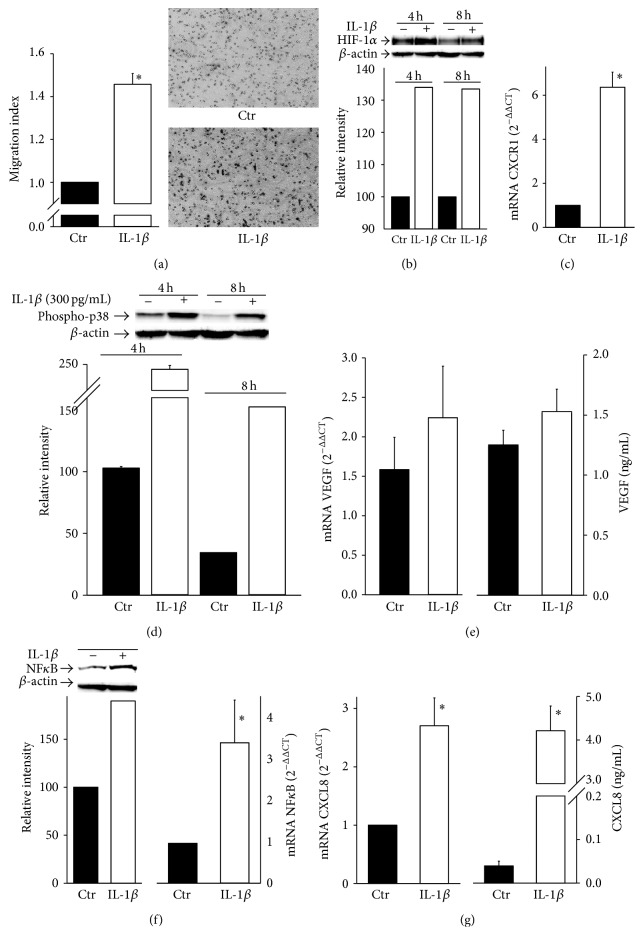
IL-1*β* increases MDAMB231 cell migratory ability under hypoxia. Data are shown as means ± SEM. ^*∗*^ *P* < 0.05, statistically significant difference between IL-1*β*-treated hypoxic cells (IL-1*β*) and hypoxic control cells (Ctr). (a) The migration assay was performed as described in Section 2. The migration index was calculated as a fold change of relative CyQuant fluorescence of cells migrating into the lower chamber normalized to that of the untreated samples (Ctr) (*n* = 4). Migrating cells were photographed and photomicrographs of representative fields from the indicated conditions were shown. (b) Protein expression of HIF-1*α* was determined by Western blot analysis at indicated time points. *β*-actin was used as loading control. A representative blot from three independent experiments is shown. Quantification of HIF-1*α* protein expression was achieved by chemiluminescence. (c) CXCR1 mRNA expression was determined by qRT-PCR analysis (*n* = 3). (d) Protein expression of phospho-p38 was determined by Western blot analysis. (e) VEGF-A mRNA and protein expression were analyzed by qRT-PCR and ELISA (*n* = 3). (f) Protein and mRNA expression of NF*κ*B was determined by Western blot and qRT-PCR analysis (*n* = 3). (g) CXCL8 mRNA and protein expression were analyzed by qRT-PCR and ELISA (*n* = 3).

We have recently reported that IL-1*β* regulates the migratory potential of MDAMB231 breast cancer cells in normoxic conditions [13]. However, given the relevance of the hypoxic microenvironment in tumor progression, we wondered how IL-1*β* might affect breast cancer cell migration under hypoxia. To this end, we employed a transwell* in vitro* assay under hypoxic conditions, where MDAMB231 cells and IL-1*β* (300 pg/mL) were added in the upper chamber of the transwell cultures. IL-1*β* concentration was selected on the base of our previous observations [13]. After 24 hours, the number of migrating cells was determined by fluorimetric analysis and, as shown in Figure 1(a), IL-1*β* significantly enhanced MDAMB231 cell migration when compared with the hypoxic controls. Such enhancement was also evident when we used another classic cell migration assay, the modified Boyden chamber assay, where cells were exposed to hypoxia, in the presence or in the absence of IL-1*β*, for only 6 hours (representative photos are shown in Figure 1(a)).

Since not only is HIF-1*α* the key regulator of hypoxia-induced adaptive responses but it is also induced by proinflammatory stimuli, we next analyzed whether HIF-1*α* accumulation under hypoxia was enhanced by IL-1*β* in MDAMB231 cells. As shown in Figure 1(b), IL-1*β* markedly enhanced HIF-1*α* accumulation under hypoxic conditions at two time points (4–8 hours). It should be underlined that, as expected in a hypoxic microenvironment, HIF-1*α* expression was also detectable in the absence of IL-1*β*, while in normoxic controls HIF-1*α* accumulation was not observed (data not shown).

IL-1*β* treatment under hypoxia resulted also in the upregulation of CXCR1, a CXC chemokine receptor actively involved in cell migration (CXCR1-CXCL8 axis) and transcriptionally regulated by HIF-1*α* (Figure 1(c)). Since p38 MAPK and VEGF-A are both important players during carcinogenesis and metastasis, we next determined their expression in hypoxic MDAMB231 in the presence of IL-1*β*. As expected, IL-1*β* enhanced the phosphorylation of p38 MAPK (Figure 1(d)). With regard to VEGF-A, its expression was apparently increased in the presence of IL-1*β*, but, when compared to the control, the difference was not significant at both mRNA and protein levels (Figure 1(e)). This may be due to the fact that VEGF-A expression was already upregulated by hypoxia even in the absence of IL-1*β* when compared to the normoxic controls (data not shown). Because IL-1*β* exerts many of its biological effects through the transcription factor NF*κ*B, we next determined whether NF*κ*B was affected by IL-1*β* in hypoxic MDAMB231 cells.  Figure 1(f) clearly shows that the phosphorylation of NF*κ*B subunit p65 was increased by IL-1*β* in hypoxic cells, as compared to hypoxic controls. This induction was confirmed also at mRNA levels, as p65 subunit mRNA expression was significantly increased in hypoxic MDAMB231 cells in the presence of IL-1*β* (Figure 1(f)).

Since CXCR1-CXCL8 ligand-receptor axis plays a critical role in cell migration [29] and CXCL8 promoter contains the binding sites for several transcription factors including NF*κ*B [30], we next determined whether IL-1*β* enhances CXCL8 expression in hypoxic MDAMB231 cells. As shown in (Figure 1(g)), when hypoxic cells were stimulated with IL-1*β*, CXCL8 expression was significantly upregulated at both mRNA and protein levels, as determined by qRT-PCR and ELISA.

This suggests that IL-1*β* promotes a migratory program in MDAMB231 cells under hypoxic conditions, by activating at least two different signaling pathways.

### 3.2. Inhibition of HIF-1*α* Does Not Affect IL-1*β*-Induced Cell Migration in Hypoxia

**Figure 2:**
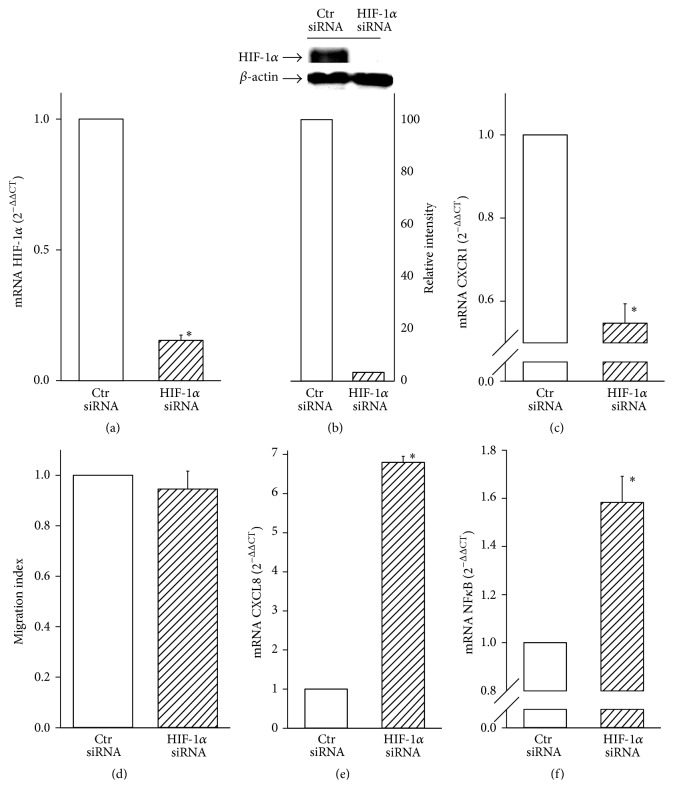
HIF-1*α* siRNA does not affect IL-1*β*-induced cell migration in hypoxia. Data are shown as means ± SEM. ^*∗*^ *P* < 0.05, statistically significant difference between IL-1*β*-treated hypoxic cells silenced for HIF-1*α* (HIF-1*α* siRNA) and IL-1*β*-treated hypoxic cells silenced with oligo controls (Ctr siRNA). (a) HIF-1*α* mRNA expression was determined by qRT-PCR analysis (*n* = 3). (b) Protein expression of HIF-1*α* was determined by Western blot analysis. *β*-actin was used as loading control. A representative blot from three independent experiments was shown. Quantification of HIF-1*α* protein expression was achieved by chemiluminescence. (c) CXCR1 mRNA expression was determined by qRT-PCR analysis (*n* = 3). (d) The migration index was calculated as a fold change of relative CyQuant fluorescence of cells migrating into the lower chamber normalized to that of the control samples (Ctr siRNA) (*n* = 4). (e and f) CXCL8 and NF*κ*B mRNA expression was analyzed by qRT-PCR (*n* = 3).

To establish the relevance of the above pathways activated by IL-1*β* in hypoxic MDAMB231 cell migration, we first investigated the role of HIF-1*α*. To this end, we inhibited HIF-1*α* expression by RNA interference. Breast cancer cells were transfected with stealth RNA oligonucleotides targeted to human HIF-1*α* mRNA (HIF-1*α* siRNA). Cells transfected with control oligos were used as controls (Ctr siRNA) and HIF-1*α* expression was analyzed to evaluate the efficacy of silencing. qRT-PCR analysis revealed that HIF-1*α* siRNA significantly inhibited mRNA expression (Figure 2(a)) and markedly decreased the protein levels of HIF-1*α* induced by IL-1*β* under hypoxia (Figure 2(b)). Because CXCR1 is transcriptionally regulated by HIF-1*α* and, as shown above, is also enhanced by IL-1*β*, we next investigated whether HIF-1*α* siRNA treatment could modify CXCR1 expression in the presence of IL-1*β*.  Figure 2(c) shows that when HIF-1 was inhibited by siRNA, IL-1*β*-induced CXCR1 mRNA expression was significantly downregulated. Thereafter, we evaluated the effects of HIF-1*α* siRNA treatment on cell migration. Unexpectedly, HIF-1*α* inhibition did not affect cell migration in the presence of IL-1*β* under hypoxic conditions (Figure 2(d)). Interestingly and in contrast with the results obtained for CXCR1 expression, such inhibition resulted in a significant increase of CXCL8 mRNA expression when compared with control siRNA (Figure 2(e)). As CXCL8 is regulated mainly by NF*κ*B, we decided to verify whether this pathway was activated by IL-1*β* under hypoxic conditions when MDAMB231 cells were silenced for HIF-1*α*.  Figure 2(f) clearly shows that NF*κ*B (p65 subunit) mRNA levels were significantly higher when compared with control siRNA, suggesting a compensatory role played by NF*κ*B under hypoxic conditions when HIF-1*α* was inhibited. In contrast, phosphorylation of p38 MAPK and VEGF expression were not significantly enhanced by HIF-1*α* siRNA (data not shown).

**Figure 3:**
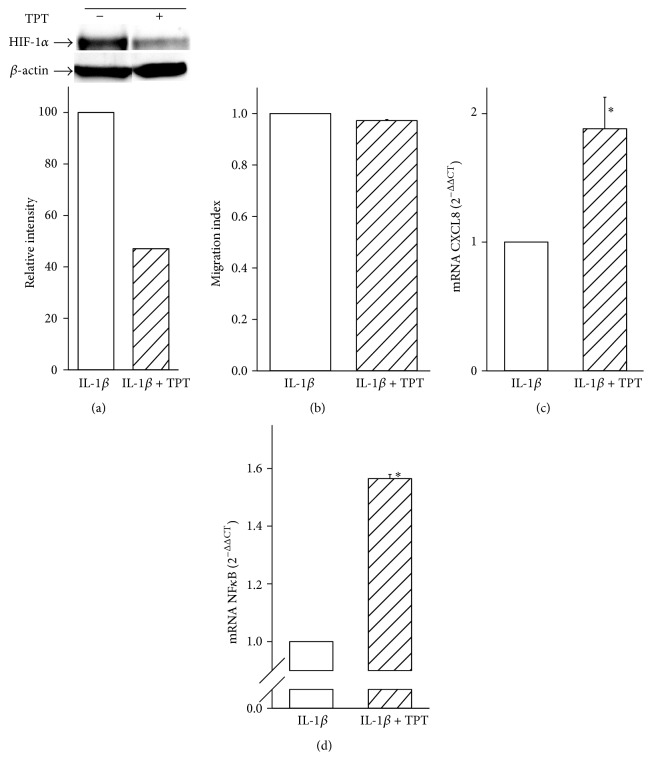
Topotecan does not reduce the induction of hypoxic breast cancer cell migration by IL-1*β*. Data are shown as means ± SEM. ^*∗*^ *P* < 0.05, statistically significant difference between IL-1*β*-treated hypoxic cells in the presence of Topotecan (IL-1*β* + TPT) versus IL-1*β*-treated hypoxic cells in the absence of Topotecan (IL-1*β*). (a) Protein expression of HIF-1*α* was determined by Western blot analysis. A representative blot from three independent experiments is shown. HIF-1*α* protein quantification was achieved by chemiluminescence. (b) Cell migration index was calculated as a fold change of relative fluorescence of cells migrating into the lower chamber normalized to that of the untreated samples (IL-1*β*) (*n* = 5). (c and d) CXCL8 and NF*κ*B mRNA expression was analyzed by qRT-PCR (*n* = 3).

To confirm that inhibition of HIF-1*α* resulted in upregulation of NF*κ*B in hypoxic breast cancer cells in the presence of IL-1*β*, we decided to perform similar experiments by using Topotecan, a topoisomerase inhibitor which has been described also as a pharmacological inhibitor of HIF-1*α* [31]. To this end, we first determined whether Topotecan was able to inhibit HIF-1*α* under hypoxic conditions in the presence of IL-1*β*.  Figure 3(a) clearly shows that Topotecan decreased the IL-1*β*-dependent accumulation of HIF-1*α* protein by ~50%. Therefore, we next explored the effect of Topotecan on breast cancer cell migration under hypoxic conditions by using the transwell* in vitro* assay. Similarly to the results obtained from HIF-1*α* siRNA experiments, treatment with 100 nM Topotecan did not affect cell migration in the presence of IL-1*β* (Figure 3(b)). In order to exclude the possibility that the lack of effect was due to either a decreased cell proliferation or cell death, we performed parallel experiments aimed at assessing cell viability. As expected, and in agreement with previous reports [32], after 24 hours 100 nM Topotecan did not significantly affect cell viability either in the absence or in the presence of IL-1*β* (data not shown). Accordingly with the HIF-1*α* siRNA results, we observed a significant enhancement of CXCL8 mRNA expression in cells exposed to hypoxia and IL-1*β* and treated with Topotecan when compared with untreated controls (Figure 3(c)). More interestingly, NF*κ*B mRNA levels were significantly upregulated by Topotecan treatment (Figure 3(d)), while, similarly to the results obtained by HIF-1*α* siRNA, phosphorylation of p38 MAPK and VEGF expression were not significantly enhanced (data not shown).

Again, such results suggest that inhibition of HIF-1*α* does not suppress the migratory program activated by IL-1*β* in hypoxic MDAMB231 cells.

### 3.3. Role of NF*κ*B in IL-1*β*-Induced Hypoxic Cell Migration

**Figure 4:**
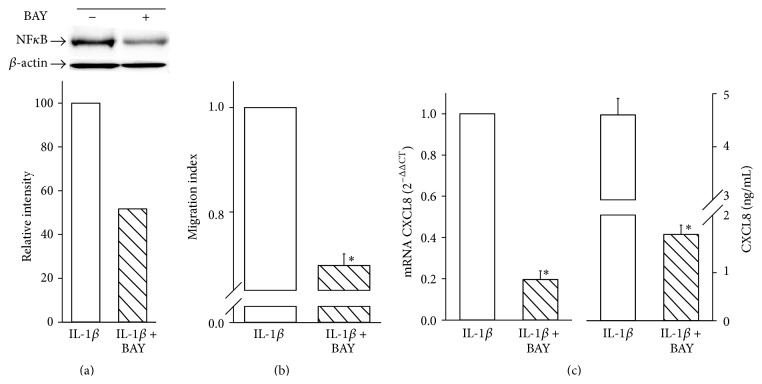
Role of NF*κ*B in IL-1*β*-induced hypoxic cell migration. Data are shown as means ± SEM. ^*∗*^ *P* < 0.05, statistically significant difference between IL-1*β*-treated hypoxic cells in the presence of BAY 11-7082 (IL-1*β* + BAY) and IL-1*β*-treated hypoxic cells in the absence of BAY 11-7082 (IL-1*β*). (a) Protein expression of NF*κ*B was determined by Western blot analysis and a representative blot from three independent experiments is shown. Quantification of NF*κ*B protein expression was achieved by chemiluminescence (*n* = 3). (b) The migration index was calculated as a fold change of relative fluorescence of cells migrating into the lower chamber normalized to that of the untreated samples (IL-1*β*) (*n* = 3). (c) CXCL8 mRNA and protein expression were analyzed by qRT-PCR and ELISA (*n* = 3).

The above results indicate that NF*κ*B appears to be functionally involved in IL-1*β*-induced cell migration under hypoxia. To further investigate the role of NF*κ*B, we next examined whether its inhibition may affect MDAMB231 cell migration induced by IL-1*β* under hypoxic conditions. To this end, we decided to employ a commercially available compound, BAY 11-7082, well known as a pharmacological inhibitor of IKK and, therefore, of NF*κ*B [33]. We first determined whether such inhibitor was able to reduce NF*κ*B levels in our experimental conditions. As shown in Figure 4(a), BAY 11-7082, at a concentration of 10 *μ*M, downregulated the expression of phosphorylated NF*κ*B p65 protein by ~50% in hypoxic MDAMB231 cells treated with IL-1*β*. More interestingly, and in contrast with results obtained either with siRNA HIF-1*α* or with Topotecan, NF*κ*B inhibition was accompanied by a significant reduction of cell migration (Figure 4(b)). Again, and as for Topotecan, at 24 hours 10 *μ*M BAY 11-7082 did not significantly affect cell viability either in the absence or in the presence of IL-1*β* (data not shown). As CXCL8 expression is regulated mainly by NF*κ*B, we next analyzed CXCL8 mRNA and protein levels in IL-1*β*-stimulated cells exposed to hypoxia and treated in the presence or in the absence of BAY 11-7082. Accordingly with the results obtained from cell migration experiments, NF*κ*B inhibition resulted in a significant decrease of CXCL8 mRNA expression (Figure 4(c)). A similar decrease was observed at protein level (from 4600 ± 312 pg/mL to 1794 ± 193).

The use of NF*κ*B pharmacological inhibitor points out not only the relevance of NF*κ*B/CXCL8 pathway in IL-1*β*-induced MDAMB231 cell migration but also a potential compensatory role with regard to HIF-1*α* in a hypoxic microenvironment.

## 4. Discussion

The data presented in this study show that the proinflammatory cytokine IL-1*β* enhances MDAMB231 cell migration under hypoxia along with an increased expression of HIF-1*α*, p65 NF*κ*B, and CXCL8/CXCR1. They also show that HIF-1*α* inhibition does not prevent the migratory program activated by IL-1*β* in hypoxic MDAMB231 cells and that such program may be preserved through NF*κ*B activation.

The tumor milieu is characterized by the presence of inflammatory cytokines and chemokines which contribute to tumor progression in a hypoxic microenvironment [2, 34]. Several studies clearly demonstrated the relevance of IL-1*β* in tumor development and metastasis [7]. IL-1*β* activates a variety of proinflammatory pathways, including NF*κ*B [35], whose relevance in cell migration has been recognized in several cell types, including breast cancer [36, 37]. In the present study we show that IL-1*β* enhances NF*κ*B p65 phosphorylation and cell migration in hypoxic MDAMB231. In addition, such enhancements were accompanied by an overexpression of CXCL8/CXCR1, whose role in tumor angiogenesis and progression has been demonstrated [29, 38]. Thus, our results support the hypothesis that IL-1*β* may activate a proinflammatory pathway associated with a promigratory program under hypoxic conditions.

A number of reports have established that IL-1*β* induces the expression of either HIF-1*α* or NF*κ*B, two of the main hypoxia sensitive pathways, which are involved in tumor progression by promoting angiogenesis, cell migration, and invasiveness [39]. Accordingly, we here show that IL-1*β* enhances HIF-1*α* accumulation in hypoxic MDAMB231 cells. It should be underlined that the expression of CXCR1 and CXCL8 is transcriptionally regulated by HIF-1*α* and NF*κ*B, respectively [13, 40, 41]. Of interest, a previous report indicated that expression of CXCL8 might preserve the angiogenic response in HIF-1-inhibited tumor cells [42], suggesting that the NF*κ*B pathway is important for the induction of CXCL8 in the absence of HIF-1*α* [42]. In our study, we report that when HIF-1*α* was inhibited, cell migration was preserved in hypoxic MDAMB231 stimulated by IL-1*β*. Indeed, IL-1*β* induces CXCL8 through NF*κ*B, while HIF-1*α* does not directly regulate CXCL8 expression.

The fact that inhibition of HIF-1*α* does not affect MDAMB231 cell migration induced by IL-1*β* under hypoxic conditions is particularly interesting when compared with our previous paper where the experiments were conducted under normoxic conditions [13]. In that study, HIF-1*α* siRNA treatment resulted in a significant reduction of IL-1*β*-induced cell migration, indicating that inhibition of HIF-*α* was able to suppress IL-1*β* effects under aerobic conditions. Thus the results shown in the present study, conducted under hypoxia, could be explained by the fact that, in conditions in which HIF-1*α* accumulation is peculiar (such as under hypoxia and in the presence of IL-1*β*), NF*κ*B may play a compensatory role, with important implications in cell migration. Once again, our results are in agreement with previous reports pointing out the relevance of NF*κ*B in tumor progression in HIF-1*α*-deficient tumors [42].

The present data, regarding the upregulation of NF*κ*B when HIF-1*α* expression is reduced, are corroborated by a recent report which unequivocally shows the intimate link between hypoxia and inflammation [43]. In that paper the authors showed that the inhibition of specific hydroxylases (e.g., PHD1 and FIH, which granted HIF-1*α* degradation under aerobic conditions) resulted in the attenuation of IL-1*β*-induced NF*κ*B-dependent gene expression. It should be recalled that such hydroxylases are inhibited under hypoxic conditions, leading to HIF-1*α* accumulation and activation of HIF-1-related responses [12].

## 5. Conclusions

Collectively, our findings underline the role of HIF-1*α* and NF*κ*B in the complexity of migration program of MDAMB231 breast cancer cells in the presence of IL-1*β* under hypoxic conditions. They also indicate that HIF-1*α* inhibition does not prevent IL-1*β*-induced cell migration under hypoxia and suggest that NF*κ*B may play a potential compensatory role to preserve their migratory capability. Finally, our results may be useful to develop new therapeutic strategies aimed at targeting differentially the inflammatory components of the multifaceted tumor microenvironment, especially in breast cancer.

## Figures and Tables

**Table 1:** List of primers used in this study for qRT-PCR.

	Forward primers 5′ to 3′	Reverse primers 5′ to 3′
CXCR1	GGATGGTGTTGCGGATCCTG	AGGGTGAATCCATAGCAGAACAG
NF*κ*B p65	TGTAACTGCTGGACCCAAGGACAT	AAAGCTGTAAACATGAGCCGCACC
CXCL8	GACATACTCCAAACCTTTCCAC	CTTCTCCACAACCCTCTGC
VEGF-A	TACCTCCACCATGCCAAGTC	ATGATTCTGCCCTCCTCCTTC
HIF-1*α*	CCAGTTACGTTCCTTCGATCAGT	TTTGAGGACTTGCGCTTTCA
*β*-actin	CGCCGCCAGCTCACCATG	CACGATGGAGGGGAAGACGG
